# Post-Ebola Syndrome among Ebola Virus Disease Survivors in Montserrado County, Liberia 2016

**DOI:** 10.1155/2018/1909410

**Published:** 2018-06-28

**Authors:** Himiede W. Wilson, Maame Amo-Addae, Ernest Kenu, Olayinka Stephen Ilesanmi, Donne K. Ameme, Samuel O. Sackey

**Affiliations:** ^1^Ghana Field Epidemiology and Laboratory Training Program, Department of Epidemiology and Disease Control, School of Public Health, College of Health Sciences, University of Ghana, Accra, Ghana; ^2^Liberia Field Epidemiology Training Program, Monrovia, Liberia

## Abstract

**Introduction:**

An increased number of survivors have emerged from the 2014 West African Ebola Virus Disease (EVD) epidemic. Post-Ebola Syndrome (PES) is a group of physical and psychological symptoms affecting EVD survivors. This study aimed to estimate the prevalence of PES among EVD survivors in Montserrado County, Liberia.

**Method:**

A cross-sectional study design was conducted to determine the prevalence of PES, types, onset, and duration among survivors. Survivors in Montserrado County were recruited using multistage sampling methods. Quantitative data was collected using semistructured questionnaire. Variables were collected on EVD survivors demographics, pre- and post-Ebola health history.

**Result:**

Prevalence of Post-Ebola Syndrome was estimated to be 90% (242/268). PES was experienced by 67% (162/242) females. PES occurred mainly in the adult population between ages 25-34, 35% (84/242). The commonest symptoms were reported from the following systems of the human body: neurological system (eyes problem, headache, sleep disorder, and unusual tiredness) and musculoskeletal system (abdominal pain, chest pain, and joints pains). The onset of PES occurred between the first 1-12 weeks after being discharged from a treatment unit.

**Conclusion:**

Prevalence of PES is high. Clinical care for survivors should be strengthened.

## 1. Introduction

The Ebola virus disease (EVD) epidemic of 2014 is the largest in history accounting for 28,646 cases as of 30^th^ March 2016 [1]. The epidemic started in December 2013 in a village called Gueckedou in the Republic of Guinea with the death of a two-year-old boy. In Liberia, the first two cases of EVD were confirmed on 30^th^ March 2014 in Foya District, Lofa County, near the border with Guinea. The number of cases of EVD reported in Liberia was 10,675 of which 4,809 died giving a case fatality rate of 45% [1].

Patients who survived EVD are faced with health conditions that may persist for a long time after being discharged from the Ebola Treatment Unit (ETU). These health conditions have been termed Post-Ebola Syndrome [PES] [2]. PES is a group of physical and psychological symptoms affecting EVD survivors. These symptoms range from short-term (12 months) to long-term [≥24 months] [3]. Some of these symptoms include headache, joint and muscles pain, extreme fatigue, menstrual cessation, clouded vision, hair loss, and deafness [4].

In Sierra Leone when the epidemic was ongoing, a study was conducted to assess survivors. More than 50% of survivors experienced symptoms such as visual problem, joint and chest pains, headaches, extreme fatigue, and depression [4]. These symptoms have made it tough for survivors to take up their prior lives for practically a year or more. Poor health seeking behavior coupled with limited access to quality healthcare and health personnel's fear complicates the management of EVD [5]. According to Schaffner (2016), the existence of poverty and poor prior nutritional status could be influencing PES. Other factors include age, viral count, EVD supportive treatment, and high concentration of disinfectant [1, 4, 6, 7].

Survivors of the 2014 West African EVD epidemic are predisposed to many health and non-health-related problems. Although the phenomenon of PES has been identified following previous EVD epidemics, due to the high case fatality rate of previous epidemics that resulted in fewer survivors, few researches were conducted. The health problems survivors experienced during recovery post-Ebola is still not fully understood. This study was conducted to document the prevalence of PES among EVD survivors in Montserrado County, Liberia.

## 2. Methods

### 2.1. Study Design

A cross-sectional study was conducted from January to April, 2016. Data was collected on the types of PES, its onset, and duration.

### 2.2. Study Area

The study was done in Montserrado County, one of the fifteen counties in Libera. It has seven health districts, namely, Careysburg, Bushrod Island, Somalia Drive, St. Paul, Central Monrovia, Todee, and Common Wealth Districts with a population of 1,287,184 inhabitants.

### 2.3. Study Population

The study population was EVD survivors recorded in Montserrado County from the beginning of the epidemic, May 2014-March 2015. Survivors in Montserrado County were 902; this accounted for 58% of survivors in Liberia. Survivors 18 years and above were 712.

### 2.4. Sample Size Determination

The sample size (n) for the study was 300 survivors. This was calculated with stat calculator in Epi Info from a population of 712 survivors line-listed by Ministry of Health (MOH), at a 50% prevalence of PES [6], a significance level of 5%, and a 20% rate for non-responses.

### 2.5. Inclusion Criteria

1. EVD survivors ≥18 years of age.
2. EVD survivor should be a resident of one of the seven health districts in Montserrado County.

### 2.6. Exclusion Criteria

1. EVD survivors without a copy or photocopy of original discharge certificate.
2. EVD survivors without a membership card of the Survivors network, Liberia.
3. An EVD survivor that refuses consent from being a part of the study.

### 2.7. Sampling Method

Survivors in Montserrado County were stratified by the seven health districts based on their location, after which, selection of survivors was done proportionately to the size of each health district. We further used a simple random number table to generate the list of participants in each health district. For participants selected, they were traced using their telephone number and interviews were conducted. Other details were previously published [8, 9].

Prior to the commencement of the study, five research assistants were hired and trained. Also, pretesting of data collection tool was carried out in both a rural and urban communities. The study protocol and instruments were approved by the University of Liberia Pacific Institute of Research and Evaluation International Review Board Monrovia, Liberia (00004982). Meetings were conducted with Ebola Survivor Network to explain the objectives and method of the study before participation of survivors. Also, a written informed consent by each participant was signed to ensure willingness, privacy, and confidentiality of information. Illiterate/incapacitated survivors were asked to thumb print using an ink pad.

### 2.8. Data Collection

Prior to the selection of participants to partake in the study, the principal researcher met with the national coordinator, president, sector heads, and sector supervisors of Ebola Survivors Network in Liberia. This meeting was organized to explain the study, its importance, and the process through which participants were to be selected to take part in the study. After which, the recruitment process of participants for the study was conducted from November-December 2015. Data collection began in January 2016 and continued until April 2016. During which time participants of the study were visited at their homes, clinics, and market places in communities around Montserrado County. Data were collected through interviews with Ebola survivors using a semistructured questionnaire to determine the prevalence of PES in Montserrado County. The questionnaire was used to record survivors demographic information, individual Ebola history, and pre- and post-Ebola medical historytory.

### 2.9. Data Analysis

Data was entered, cleaned, coded, and analyzed with SPSS version 23.

The characteristics of study participants were explained through descriptive statistics; continuous variables were presented as median and range. Categorical variables were presented in composite tables showing frequencies and percentage distributions. Graph and charts were also used to present part of the data.

### 2.10. Definition of Terms

 Post-Ebola Syndrome. Health-related problems that occurred in EVD survivors after laboratory result has proven negative.

 Short-Term PES. Health-related symptoms that manifest between 1 and 10 months after discharge from an ETU.

 Survivors. A patient subsequently recovers, after being confirmed positive with a result of RT-PCR testing for Ebola virus on anybody fluid (“WHO | Ebola virus disease,” 2015).

## 3. Results

Table 1 shows the distribution of sociodemographic characteristics of all participating EVD survivors. Sixty-five percent (174/268) of the study participants were females. For the 252 respondents who could recall their ages during the interview, median age was 30 years, and the age ranged from 18 to 70 years. Most of the participating EVD survivors in the study were single accounting for 62% (166/268), followed by married survivors accounting for 25% (67/268). Of the total participating survivors, 59% (158/268) were not employed. Also of all respondents who stated their occupations, 27% (73/268) engaged in business and 18% (48/268) were students.

Of all respondents, 9.7% (26/268) had never experienced PES. The remaining 90.3% (242/268) complained of at least having experienced a health problem.

Table 2 shows the prevalence of PES among EVD survivors. It was observed that PES occurred mainly in the adult population between ages 25-34 years 91% (84/92) and 35-44 years 92% (76/83). Females with PES were 162 (93%) compared to 80 (85%) male counterparts. The prevalence of PES was 91% (151/166) among the single respondents and 97% (30/31) for widower/widow. Also, the prevalence of PES among EVD survivors that were unemployed was 87% (138/158).

The commonest symptoms observed among respondents with PES were joint pains 64% (154/242), headache 52% (127/242), eyes problem 47% (114/242), muscles pain 34% (80/242), and unusual tiredness 28% (68/242). The types of PES reported among respondents are shown in Table 3.

“Others” represent symptoms other than the 14 listed above which includes absentmindedness, hair loss, generalized body pain, back aches, swollen feet, diabetes, hemorrhoids, ear pain, hearing loss, erectile dysfunction, heart palpitation, frequent fever, numbness of feet, liver, and heart problems. Some survivors interviewed did not have PES; hence, symptoms did not add up to 268.

**Figure 1:**
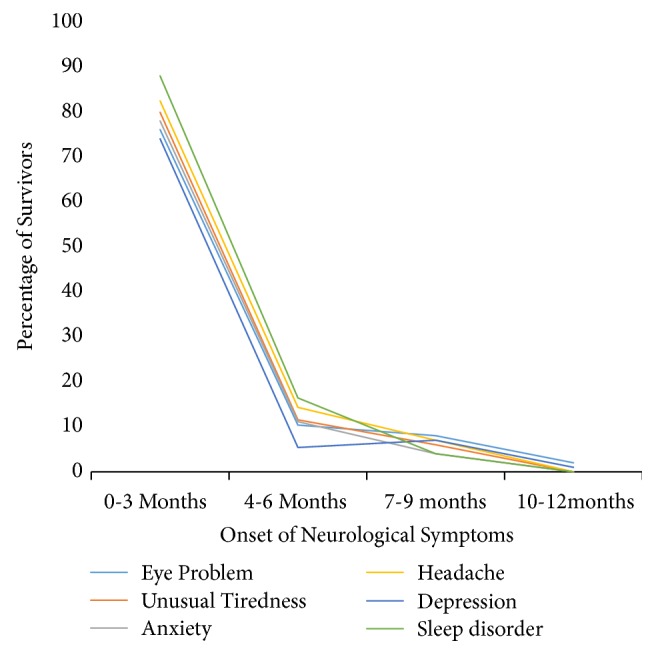
Onset of PES of the neurological system among EVD survivors in Montserrado County, Liberia, 2015-2016.

**Figure 2:**
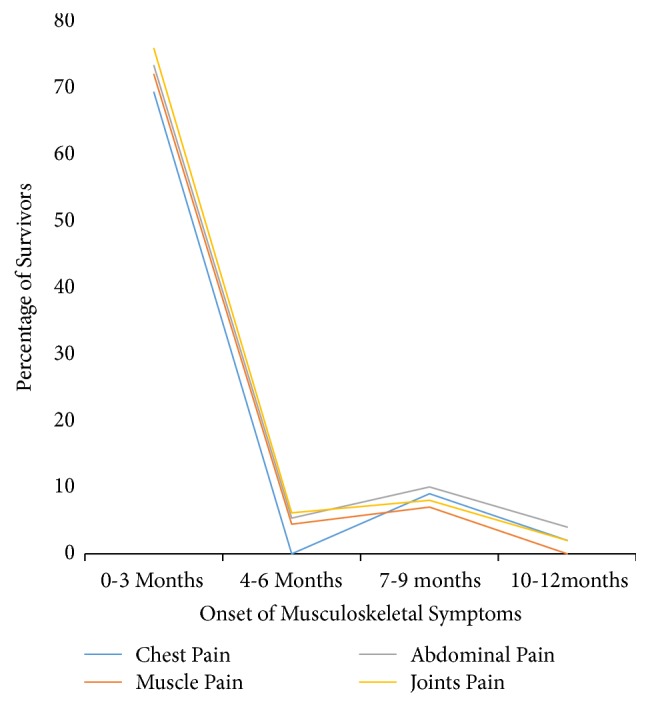
Onset of PES of the musculoskeletal system among EVD survivors in Montserrado County, Liberia, 2015-2016.

Within the first 3 months of recovery from EVD, the majority of survivors started experiencing musculoskeletal symptoms such as joint pain 77% (118/154), muscles pain 68.2% (60/88), chest pain 67% (38/57), and abdominal pain 62%, (38/61). In the second quarter 4-6 months symptoms with onset were chest pain 21% (12/57) of survivors, abdominal pain 19.7% (12/61), joints pain 16% (25/154), and muscles pain 17% (15/88). Symptoms with onset between 7 and 9 months were abdominal pain accounting for 13% (8/61), chest pain 8.8% (5/57), muscles pain 8% (7/88), and joints pain 0.6% (1/154). As reported in the last quarter with onset between 10 and 12 months, muscles pain accounting for 6.8% (6/88), joints pain 6.5% (10/154), chest pain 8.8% (2/57), and abdominal pain 4.9% (3/61) were reported.  Figure 1 shows the onset of PES of the neurological system and Figure 2 shows the onset of PES of the musculoskeletal system.

Table 4 shows the onset of PES of neurological origin. During the first 3 months of recovery from EVD, majority of EVD survivors had started experiencing a wide range of neurological problems consisting of headache 85.8% (109/127), depression 75% (30/40), eyes problems 75% (86/114)%, sleep disorder 75% (43/57), unusual tiredness 75% (51/68), and anxiety 60.8% (28/46). By the second quarter 4-6 months less number of survivors started experiencing neurological symptoms anxiety 32.6% (15/46), eyes problem accounting for 16.7 (19/114), unusual tiredness 16% (11/68), depression 12.5% (5/40), sleep disorder 14% (8/57), and headache 9% (12/127) and sleep disorder 14% (8/57). Between the 7 and 9 months of symptoms onset, depression accounted for 7.5% (3/40), anxiety 4% (2/46), sleep disorder 3.5% (2/57), and unusual tiredness 3% (2/68), while between 10 and 12 months fewer survivors started complaining of neurological symptoms such as eyes problems accounting for 7.9% (9/114), headache 4.7% (6/127), unusual tiredness 6% (4/68), depression 5% (2/40), headache 4.7% (6/127), anxiety 2% (1/46), and sleep disorder 1.8% (1/57).

Testes pain was reported by 53.8% (7/13) of survivors to have started during the first 3 months after discharge; 30.7% of male survivors reported testis pain to have started by 4-6 months while during 7-9 months, testis pain was accounted for by 15.4% (2/13) of male survivors. Testis pain was not reported with onset between 10 and 12 months. Among the females survivors, 91% (29/32) had menstrual problems with onset beginning the first 3 months after discharge, while 9.4% (3/32) stated menstrual problem to have started by 10-12 months. Menstrual problems were not reported by female survivors to have started between 4 and 9 months. The menstrual problems complained of included menstrual irregularities and cessation.

Itching of the skin were reported by 86% (31/36) of survivors to have started by the first 3 months, 8.3% (3/36) stated their skin started itching by the 4-6 months after discharged, while 5.5% (2/36) said they started experiencing symptoms between 7 and 9 months. Peeling of the skin was reported by 89% (41/46) of survivors between the first 3 months, while 8.7% (4/46) said they started experiencing skin itching by 6 months and 2% (1/46) with symptom onset by 9 months.

## 4. Discussion

Every nine out of ten survivors complained of having PES after being discharged from the ETU in Montserrado County, Liberia, and this conforms with Qureshi 2015 study which reported close to 100% symptoms in Guinea [10]. A study by Nabena et al. recorded PES in more than half of the survivors in Sierra Leone. However, the study by Nabena was done in less than six months after discharge from an ETU, while our study was done 12 months after discharge. Occurrence of PES increases with time after discharge from ETU.

Females account for the majority of our study participants. In Liberia, the higher number of females participants could be attributed to females constituting more than half of survivors in the country. Other studies have also reported more female survivors [11, 12]. The proportion of females admitted was more than males.

Our findings conform to other studies, which showed that the majority of survivors had reported more than one symptom [11, 12]. The frequently reported symptoms recorded in this study were eye complaint, arthralgia, peeling of skin, and headaches. Other symptoms included psychological problem, chest and muscles pain, testes pain, skin diseases, hair loss, abdominal pain, ear complaint, menstrual problem, and unusual tiredness, some of which appeared after the onset of EVD infection in patients. Due to the prevalence of malaria and other infections that resemble EVD in Liberia, prior symptoms similar to EVD could not be differentiated by survivors [11, 13]. Also, preliminary findings of survivors in Liberia by PREVAIL recorded frequently experienced symptoms such as weakness, headache, memory loss, depressed mood, and muscles pain [14].

PES was mostly experienced among the age categories of adults between ages 25-34 and 35-44 years. These are mostly people who work to earn their living whether through skilled or unskilled jobs in Liberia. Consistent with our study was a study in Sierra Leone which stated that many adult survivors reported health problems and their justification was that adults mostly perform work that demanded energy [11].

Though pain exists without any activity in few survivors, pain intensifies when trying to carry out normal day to day activities like selling, walking distances, and doing house chores. The preliminary findings of a five-year clinical trial conducted in Liberia also recorded that joint pain was reported among approximately fifty percent of survivors visiting the Medicine San Frontier Clinic. Joints pain was described as pain in the elbows, wrist, fingers, hips, ankles, neck, back, and knee [3].

Ocular problems occurred in less than 50% of EVD survivors. The ocular problems were blurred vision, redness, pain, and itching of eyes. In Sierra Leone, two weeks after being discharged, 14% of survivors reported ocular problems which ranged from eye pain, eye discharge, red eyes, and blurred vision [12]. In the United States, 23% reported eye pain, blurred eyes site between two to eight weeks after being discharged from an ETU. Also in three studies in Sierra Leone, ocular complications were reported by 34%, 57%, and 60% of EVD survivors, respectively [11, 15, 16]. Survivors receiving care at the Medicine San Frontier Clinic in Liberia suffered ocular complications ranging from eye pain, inflammation of the uvea, photophobia, loss of visual acuity, and hyperlacrimation [3].

A range of neurological disorders were reported including headache which accounted for 50%. Headache was one of the most commonly reported symptoms [17]. These were initial findings within six months after being discharged from an ETU. The study did not establish as to whether these neurological symptoms were associated with severe EVD infection [3]. Headache was experienced by 48% of EVD survivors in a study in Sierra Leone which was termed as pain affecting the full head [12]. Another study in Sierra Leone showed that more than 70% of EVD survivors reported headache [11]. Other neurological symptoms reported by EVD survivors in this study were unusual tiredness, anxiety, depression, sleep disorder, absentmindedness, and numbness of hands and feet..

Out of the total EVD survivors that participated in this study, 26% suffered unusual tiredness. Thirty-two percent of EVD survivors enrolled in the clinical research had also experienced unusual tiredness, while almost 80% of EVD survivors in the United States experienced unusual tiredness [18].

Respondents complained of depression in this study and this was consistent with other studies that reported 15% of EVD survivors had suffered depression. Also, a study in the United States shows that about half of EVD survivors experienced depression [18]. Sleep disorder was experienced by 20% of survivors in this study, while more than half of EVD survivors in the United States suffered the same [18] and less than 25% poor sleep cases were reported by two studies in Sierra Leone [12, 15].

Ear problem was reported in a small number of less than two percent of EVD survivors in this study. Ear problems as described by this study include hearing loss and ear pain. Consistent with these findings was a literature in Sierra Leone which stated that a little more than five percent of EVD survivors experienced ear problems [11]. Another study in Sierra Leone found that almost 25% of EVD survivors suffered ear problems [16], while ear problems were reported among 12% of EVD survivors in the United States [18].

Skin diseases were reported by 15% of survivors and desquamation of the palm and feet has been reported by 18% of EVD survivors. The findings in this study conform with reports by the Medicine San Frontier Clinic in Liberia which stated that EVD survivors reported experiencing desquamation and dryness of skin which commonly affected the palm and soles of the feet [3]. Also stated by two previous studies in Sierra Leone, itching of the skin was reported by 9% of EVD survivors [12] Hair loss was experienced by 8% of the respondents in our study.

Less than 10% EVD survivors in this study reported reproductive system complications that include erectile dysfunction and testes pain. Female survivors also experienced menstrual problems including menstrual cessation and irregularities which accounted for 11% of female survivors. Consistent with these findings is Nabena et al. study from Sierra Leone which reported that erectile dysfunction and menstrual problems accounted for less than 5% each in the population of EVD survivors studied [11].

Finding from this study revealed that the onset of the symptoms was between the first 1 and 12 weeks after discharge from an ETU. Our finding was consistent with other studies which stated that symptoms onset began immediately after discharge or the first few weeks after [3, 11, 12, 15]. Also, literature in the United States reported that onset of new symptoms in EVD survivors was 10 weeks after discharge [19]. It was also indirectly stated by a study in Sierra Leone that PES are usually common during the early convalescence stage of EVD [16]. A survivor in Nigeria reported she left the ETU with some symptoms that persisted after discharge [2].

In this study, testicular pain and itching of the skin were less common among EVD survivors by the ninth month with less than three and ten cases recorded, respectively. Other symptoms reported by fewer EVD survivors by >10 month were menstrual problem, chest pain, abdominal pain, and depression with 17, 12, 19, and 14 percent, respectively. While symptoms that were most likely to have persisted above >10 months were eyes problems (53%), joints pain (40%), muscles pain (28%), unusual tiredness (38%), and headache (80%). This is consistent with literature published in the Democratic Republic of Congo which stated that joint pains, muscles pain, abdominal pain, and fatigue were the commonest reported during a six-month follow-up visit, while muscles and joints pain remained high among EVD survivors up to the twenty-one months after EVD [20].

A cohort study in Uganda revealed long-term symptoms that might continue for greater than two years. Commonest symptoms reported by this cohort were blurred vision, hearing loss, difficulty in swallowing, joint pains, and sleep disorders [21]. Commonest symptoms recorded tend to persist in this cohort which is a bit different from findings of our study.

We were not able to compare respondents with PES to those without PES. This is a limitation of this study given that some respondents found it difficult to discriminate true PES occurrences from other complaints not related to PES in the study population.

## 5. Conclusion

This study indicated that the prevalence of Post-Ebola Syndrome among EVD survivors in Montserrado County, Liberia, is high. The onset of symptoms was reported between the first 1 and 12 weeks after discharged from the ETU. Commonest symptoms reported were chest pain, muscle pain, eyes problem, abdominal pain, joint pain, unusual tiredness, sleep disorder, and headache. Symptoms among survivors were often intermittent and peaked by the sixth month. By the ninth month, decline was observed in the number of symptoms reported. Notwithstanding, a few number of symptoms were shown to have persisted among survivors above ten months but with reduced severity, such symptoms include eyes problem, joints pain, unusual tiredness, and headache. Clinical care for survivors in Montserrado County remains the biggest gap; very few of survivors have received appropriate treatment for complaints suffered.

## 6. Recommendations

Based on the findings of this study, the following recommendations were made.

### 6.1. Ministry of Health

The Ministry of Health, Liberia, negotiated with partners (World Health Organization, Center for Disease and Control, and Medicine San Frontiers) to improve and maintain the care being offered to survivors by increasing the number of health care centers for survivors, hiring of specialist for the commonest reported conditions, and the building of laboratory capability for advanced testing and screening. The implementation of the WHO 2016 clinical guide by care givers at the ETU to schedule preliminary follow-up visits at clinics for survivors upon leaving the ETU before the country was declared free of EVD was ensured. This enables adequate monitoring of survivors recovery status and immediate detection of any deviation in health post-EVD to avoid long-term disabilities.

## Figures and Tables

**Table 1:** Distribution of sociodemographic characteristics of all participating EVD survivors in the study in Montserrado County, Liberia, 2015-2016.

Variables	N=268	%
**Age Categories (years)**		
18-24	47	18
25-34	92	34
35-44	83	31
45-54	34	12.7
55-64	5	2
>65	1	0.3
Unknown*∗*	6	2
**Sex**		
Male	94	35
Female	174	65
**Marital status**		
Single	166	62
Married	67	25
Divorced	4	1
Widower & Widow	31	12
**Employment status**		
Employed	107	40
Unemployed	161	60

Unknown*∗* depicts survivors who could not recall their ages during the interview.

**Table 2:** Prevalence of PES among EVD survivors by age, sex, and marital and employment status in Montserrado County, Liberia, 2015-2016.

**Variables**	**Yes**	**No**
	**(N=242)**	**(N=26)**
	n(%)	n(%)
**Age Categories (years)**		
18-24	40(85)	7(15)
25-34	84(91)	8(9)
35-44	76(92)	7(8)
45-54	31(91)	3(9)
≥55	5(83)	1(17)
Unknown ages	6(100)	0
**Sex**		
Male	80(85)	14(15)
Female	162(93)	12(7)
**Marital status**		
Single	151(91)	15(9)
Married	57(85)	10(15)
Divorced	4(100)	0
Widower/Widow	30(97)	1(3)
**Employment status**		
Employed	104(95)	6(5)
Unemployed	138(87)	20(13)

*∗*yes: EVD survivors having PES; *∗*no: EVD survivors who did not report having PES; and n: number of people.

**Table 3:** Types of PES reported among respondents in Montserrado County, Liberia, 2015-2016.

** PES (n=242)**	**Frequency**	**Percentage **(%)
Chest pain	57	**23.5**
Muscles Pain	88	**36.3**
Eyes Problems	114	**47.0**
Abdominal Pain	61	**25**
Testis Pain*∗*	13	16.2
Joints Pain	154	**63.6**
Menstrual Problems*∗∗*	32	19.7
Unusual Tiredness	68	**28**
Itching of Skin	39	16
Peeling of the Skin	46	19
Anxiety	46	19
Depression	40	16.5
Sleep Disorder	57	**23.5**
Headache	127	**52**
Others	181	74.7

*∗* is percentage calculated only for male.*∗∗* is percentage calculated only for female.

**Table 4:** Duration of Post-Ebola Syndrome among EVD survivors in Montserrado County, Liberia, 2015-2016.

Symptoms	Duration							
	0-3 months		4-6 months		7-9 months		10-12 months	
**Neurological**	(n)	%	n	%	n	%	n	%
Sleep disorder	10	17.5	10	17.5	15	26	22	38.6
Unusual tiredness	14	20.6	5	7.4	11	16.2	38	55.9
Anxiety	23	50	15	32.6	4	8.7	4	8.7
Headache	26	20.5	10	7.9	11	8.7	80	62.9
Eye problem	36	31.6	15	13.2	8	7	55	48
Depression	18	45	6	15	2	5	14	35

**Musculoskeletal**	(n)	%	(n)	%	(n)	%	(n)	%
Chest Pain	28	48.3	5	8.6	13	22	12	20.7
Joints Pain	20	12.7	23	14.6	40	25.3	75	47.5
Muscles Pain	34	38.6	12	13.6	14	15.9	28	31.8
Abdominal	29	47.5	7	11.5	6	9.8	19	31.1

**Reproductive**	(n)	%	(n)	%	(n)	%	(n)	%
Testis Pain	2	15.4	6	46.2	4	30.8	1	7.7
Menstrual Problems	8	25	4	12.5	3	9.4	17	53

**Integumentary**	(n)	%	(n)	%	(n)	%	(n)	%
Itching of skin	24	66.7	12	33	3	8.3	1	2.8
Peeling of skin	36	78.3	8	17.4	2	4.3	1	2.2

## Supplementary Materials

Supplementary Materials Annex 1: assessing Post-Ebola Syndrome in survivors.
