# Supplementary material for: Post-Ebola Syndrome among Ebola Virus Disease Survivors in Montserrado County, Liberia 2016
Source: Biomed Res Int. 2018 Jun 28;2018:1909410. doi: 10.1155/2018/1909410 (PMC6046154; doi:10.1155/2018/1909410)
Supplement: Supplementary Materials — Annex 1: assessing Post-Ebola Syndrome in survivors. [file 1909410.f1.docx]

**Annex 1**

**Assessing Post-Ebola Syndrome in Survivors**

Questionnaire Number ___________

Interview Date ____/____/_____ (dd-mm-yyyy)

**Background Information**

| 1. | Sex of Respondent | [ ] 1). Male  [ ] 2).Female |
| --- | --- | --- |

| 2. | How old are you as of your last birthday | _________/____________  Years Month/Days |
| --- | --- | --- |

| 3. | Marital status | [ ] 1). Single  [ ] 2). Married  [ ] 3).Divorced  [ ] 4).Widow/Widower |
| --- | --- | --- |

| 4. | Where do you live? |  |
| --- | --- | --- |

| 5. | Education level | [ ] 1). Primary [ ] 2). Secondary  [ ]3).Tertiary [ ] 4). Others(specify) |
| --- | --- | --- |

| 6. | Are you employed?  If no skip question 7 | [ ] 1).Yes [ ] 2).No |
| --- | --- | --- |

| 7. | Occupation | [ ] 1). Nurse  [ ] 2). Farmer  [ ] 3). Teacher  [ ] 4). Businessman/Woman  [ ] 5).Carpenter  [ ] 6). Others (specify) |
| --- | --- | --- |

**Ebola History**

Now we would like to get some brief information regarding when you became sick and the dates that you were in the hospital. If you do not know the exact day, you can guess. If you only know the month, that is fine also. For all dates please use the dd/month format using a 3 LETTER abbreviation for month e.g. 02/SEP/2009)

| 8. | What date/month you started feeling sick? | _________ /__________/________  Day Month Year |
| --- | --- | --- |

| 9. | Which date did you reach a hospital? | _________/__________/_________  Day Month Year |
| --- | --- | --- |

| 10. | Which Ebola treatment center were you admitted to? | [ ] 1). ELWA 2  [ ] 2). ELWA 3  [ ] 3).MOD 1  [ ] 4). MMU  [ ] 5).JFK  [ ] 6). UCC  [ ] 7). CHINA  [ ] 8).ISLAND clinic  [ ] 9).BONG |
| --- | --- | --- |

| 11. | Which date were you discharged? | ________/_________/___________  Day Month Year |
| --- | --- | --- |

| 12. | How long did you stay in the ETU? | _____________/________________  Week(s) Month(s) |
| --- | --- | --- |

| 13. | During your stay in the ETU, do you remember the drugs given you? | [ ] 1).Yes [ ] 2).No |
| --- | --- | --- |

| 14. | What were the drugs? Please specify. | [ ] 1).Paracetamol (pain Killer)  [ ] 2).ORS  [ ] 3).Antibiotics  [ ] 4).Antimalarial Pills  [ ] 5).Others |
| --- | --- | --- |

**15. Pre and Post Ebola Medical History**

(Now we would like you to tell us what medical conditions you had BEFORE and AFTER Ebola to see how it compares). In the above question, you told us you got sick with Ebola on XX (look at Date #7). BEFORE this date, can you please tell us what medical problems you had?

|  | **Symptom** | **Before** | **After**  **(**Can you please tell us what problems you are facing now?)**.** |
| --- | --- | --- | --- |
|  | Chest pain |  |  |
|  | Muscle pain |  |  |
|  | Eyes problem |  |  |
|  | Stomach pain |  |  |
|  | Pain in the testis |  |  |
|  | Joint pain |  |  |
|  | Menstrual problem |  |  |
|  | Unusual tiredness |  |  |
|  | Itching of skin |  |  |
|  | Peeling of skin |  |  |
|  | Worried/frightened |  |  |
|  | Depression |  |  |
|  | Failure to sleep |  |  |
|  | Headache |  |  |
|  | Others? Specify |  |  |

16) Can you please indicate which month after your discharge you started experiencing symptom?

|  | **Symptom** | **Onset of PES** | | | |
| --- | --- | --- | --- | --- | --- |
|  |  | **Months 0-3** | **Months 4- 6** | **Months 6- 9** | **Months 10-12** |
| a) | Chest pain |  |  |  |  |
| b) | Muscle pain |  |  |  |  |
| c) | Eyes problem |  |  |  |  |
| d) | stomach pain |  |  |  |  |
| e) | Pain in the testis |  |  |  |  |
| f) | Joint pain |  |  |  |  |
| g) | Menstrual problem |  |  |  |  |
| h) | Unusual tiredness |  |  |  |  |
| i) | Itching of skin |  |  |  |  |
| j) | Peeling of the skin |  |  |  |  |
| l) | Worried/frightened |  |  |  |  |
| m) | Depression |  |  |  |  |
| n) | Failure to sleep |  |  |  |  |
| o) | Headache |  |  |  |  |
| p) | Others(specify) |  |  |  |  |

**17.** (Now we would like to know the duration of the symptoms you have mentioned in (ques 16) in the **after** column above. You will be assessed on a quarterly basis for 12 months).

|  | **Symptom** | **Duration of PES** | | | |
| --- | --- | --- | --- | --- | --- |
|  |  | **Months 0-3** | **Months 4- 6** | **Months 6- 9** | **Months 10-12** |
| a) | Chest pain |  |  |  |  |
| b) | Muscle pain |  |  |  |  |
| c) | Eyes problem |  |  |  |  |
| d) | stomach pain |  |  |  |  |
| e) | Pain in the testis |  |  |  |  |
| f) | Joint pain |  |  |  |  |
| g) | Menstrual problem |  |  |  |  |
| h) | Unusual tiredness |  |  |  |  |
| i) | Itching of skin |  |  |  |  |
| j) | Peeling of the skin |  |  |  |  |
| l) | Worried/frightened |  |  |  |  |
| m) | Depression |  |  |  |  |
| n) | Failure to sleep |  |  |  |  |
| o) | Headache |  |  |  |  |
| p) | Others(specify) |  |  |  |  |

| 18. | Have you received any treatment for any of the conditions above? | [ ] 1). Yes [ ] 2). No |
| --- | --- | --- |

| 19. | If yes, can you list the drugs given you? |  |
| --- | --- | --- |

| 20. | Did you received any improvement from the drugs given you? | [ ] 1).Yes [ ] 2). No |
| --- | --- | --- |

| 21. | If no to question 16, why you didn’t received any treatment? | [ ] 1). No money  [ ] 2). Fear of stigmatization  [ ] 3).Distance to Hospital  [ ] 4). Others (specify) |
| --- | --- | --- |

| 22. | Please explain to what extend is the problem affecting your normal life? Can you still work? |  |
| --- | --- | --- |

| 23. | Are these symptoms affecting your personal relationship? Please explain how? |  |
| --- | --- | --- |

| 24. | Upon discharged from ETU back to your community, how were you received? (Here we address issue of stigmatization). |  |
| --- | --- | --- |

| 25. | Is there anything you would like me to know that I have not ask you? If yes please explain? |  |
| --- | --- | --- |

**Thanks for your Cooperation**
